# Controlled synthesis of uniform multilayer hexagonal boron nitride films on Fe_2_B alloy

**DOI:** 10.1039/c9ra00595a

**Published:** 2019-04-01

**Authors:** Zhiyuan Shi, Guangyuan Lu, Peng Yang, Tianru Wu, Weijun Yin, Chao Zhang, Ren Jiang, Xiaoming Xie

**Affiliations:** State Key Laboratory of Functional Materials for Informatics, Shanghai Institute of Microsystem and Information Technology, Chinese Academy of Sciences Shanghai 200050 China trwu@mail.sim.ac.cn; School of Electronic, Electrical and Communication Engineering, University of Chinese Academy of Sciences 19 A Yuquan Road Beijing 100049 China; CAS Center for Excellence in Superconducting Electronics (CENSE) Shanghai 200050 China; State Key Laboratory of ASIC and System, School of Information Science and Technology, Fudan University 220 Handan Road Shanghai 200433 China; State Key Laboratory of Precision Spectroscopy, School of Physics and Material Science, East China Normal University 3663 N. Zhongshan Road Shanghai 200062 China; School of Physical Science and Technology, ShanghaiTech University Shanghai 200031 China

## Abstract

Two-dimensional (2D) hexagonal boron nitride (h-BN) is highly appreciated for its excellent insulating performance and absence of dangling bonds, which could be employed to maintain the intrinsic properties of 2D materials. However, controllable synthesis of large scale multilayer h-BN is still very challenging. Here, we demonstrate chemical vapor deposition (CVD) growth of multilayer h-BN by using iron boride (Fe_2_B) alloy and nitrogen (N_2_) as precursors. Different from the self-limited growth mechanism of monolayer h-BN on catalytic metal surfaces, with sufficient B source in the bulk, Fe_2_B alloy promotes the controllable isothermal segregation of multilayer h-BN by reacting with active N atoms on the surface of the substrate. Microscopic and spectroscopic characterizations prove the high uniformity and crystalline quality of h-BN with a highly orientated layered lattice structure. The achievement of large scale multilayer h-BN in this work would facilitate its applications in 2D electronics and optoelectronics in the future.

## Abstract 2

CVD growth of large scale and high quality multilayer h-BN.

## Graphical abstract

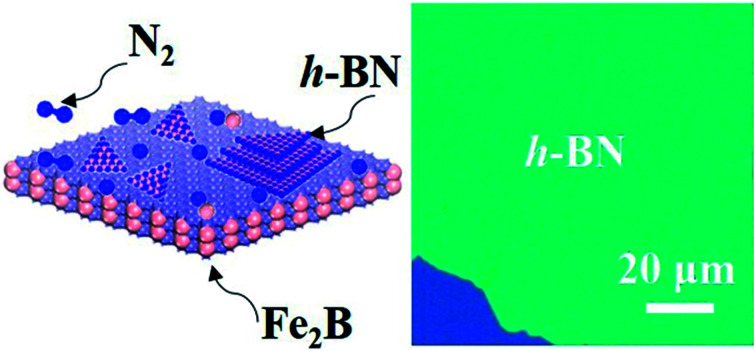

## Introduction

Two-dimensional (2D) materials have attracted enormous attention for future electronic and optoelectronic device integrations.^1–3^ Hexagonal boron nitride (h-BN), a 2D insulator with a wide band gap (∼6 eV), is in general highly desired for practical device applications, such as gate dielectric layers, tunnelling barriers or encapsulations.^4–7^ It is widely regarded as an ideal dielectric substrate for 2D materials because of its atomic flatness and absence of dangling bonds, which could maintain the intrinsic properties of the overlying 2D materials.^3,5,6,8^ Moreover, single phonon emission from isolated point defect sites in h-BN has been investigated recently, which demonstrates potential in quantum photonics and quantum computation.^9–11^

So far, many efforts have been devoted to synthesize high-quality h-BN films. In recent years, layered h-BN flakes, integrated with other layered materials for 2D heterostructure devices, were mechanically exfoliated from high quality h-BN bulk crystal, which was obtained under extremely high temperature and pressure for several days.^12,13^ However, the limited size and inhomogeneous thickness hinder further development of 2D electronics and photonics. As the most promising technique, chemical vapor deposition (CVD) was employed to achieve large-area monolayer and few layer h-BN on metal or dielectric substrates by using ammonia borane (NH_3_·BH_3_) or borazine (B_3_N_3_H_6_) as the precursor.^14–23^ However, the inhomogeneous thickness, poor crystallinity, toxicity of precursors and complicated procedures limits the application in practical 2D devices. High quality, thickness uniformity, low cost and facile synthesis remain extremely challenging for compound 2D layered materials such as h-BN.

Here, we report a new method to synthesize multilayer h-BN on Fe_2_B alloy with N_2_ as a reactant. It is much easier to control the flow rate by using gaseous N_2_ as a precursor than solid precursors (NH_3_·BH_3_ or B_3_N_3_H_6_). Comparing with previous approaches, the new synthesis method yields large scale and high quality multilayer h-BN at ambient pressure. The surface morphology, crystalline quality and defect distributions were investigated by various characterizations. Fe_2_B, an alloy not only supplies boron source directly, but also reacts with N_2_ to form multilayer h-BN. The entire growth process breaks the traditional self-limited growth mechanism on catalytic metal substrates and successfully obtains multilayer h-BN on Fe_2_B alloy. Controllable synthesis of h-BN film establishes a significant approach for further rational catalyst design for other compound 2D materials.

## Materials and methods

Multilayer h-BN was synthesized on Fe_2_B alloy using ambient pressure CVD. Firstly, a Fe_2_B substrate was heated to 1300 °C and maintained for 60 minutes under Ar and H_2_ atmosphere at flow rates of 300 and 50 sccm, respectively. Then, N_2_ and H_2_ atmosphere at flow rates of 300 sccm and 50 sccm were introduced at 1300 °C for 60 minutes to facilitate h-BN thin film growth. Finally, the furnace was cooled down to room temperature naturally under Ar and H_2_ flows.

The as-grown multilayer h-BN films were transferred to arbitrary substrates through chemical transfer process. The morphology of multilayer h-BN on Fe_2_B alloy was characterized by scanning electron microscope (SEM, Zeiss Supra 55, operated at 3 kV). Atomic force microscope (AFM, Bruker Icon, Tapping mode) was employed to determine the thickness of multilayer h-BN. Raman spectra were obtained in the spectral range of 1100–1700 (cm^−1^) by using a laser excitation of 532 nm. The stoichiometry of B and N of h-BN was evaluated by X-ray photoemission spectroscopy (XPS, species, Al Kα). The phase structure of h-BN thin film was identified by X-ray diffraction (XRD, Bruker discover D8). Transmission electron microscope (TEM, JEM-2100F, operated at 200 kV) images and selected area electron diffraction (SAED) confirm the high crystalline quality of as-prepared h-BN. Photoluminescence (PL) mapping and PL spectra were collected by using a laser excitation of 532 nm (WITec Alpha 300R, 5 mW).

## Results and discussion

**Fig. 1:**
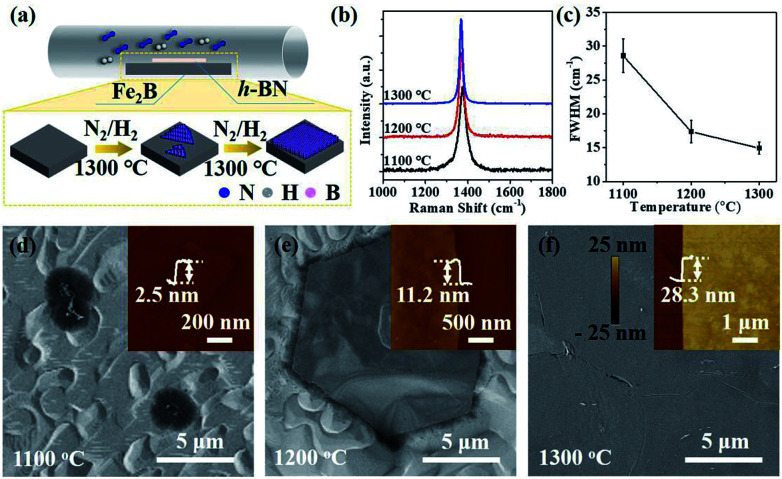
(a) Schematic diagram for synthesis of multilayer h-BN on Fe_2_B alloy. (b) Raman spectra of h-BN grown on Fe_2_B alloy at 1100 °C, 1200 °C and 1300 °C, respectively. (c) The relationship between FWHM of E_2g_ phonon mode and growth temperatures. (d–f) SEM images of h-BN grown on Fe–B alloy at 1100 °C (d), 1200 °C (e) and 1300 °C (f), respectively. Corresponding AFM images with height profiles were displayed in the inset.

The schematic of ambient-pressure CVD setup for multilayer h-BN growth was depicted in Fig. 1a (details are given in Materials and methods). To demonstrate the growth dynamics, Fe_2_B alloys were annealed at 1100 °C, 1200 °C and 1300 °C for 60 minutes, respectively. SEM images exhibit h-BN nucleus, isolated h-BN flakes and continuous h-BN film synthesized at different growth temperatures (Fig. 1d–f). Corresponding AFM images and height profiles in the inset verify the thickness of as-grown h-BN transferred on SiO_2_ (300 nm)/Si substrate. By introducing N_2_ gas as a reactant, uniform multilayer h-BN films over large areas formed on Fe_2_B substrate. Fig. 1b displays the Raman spectra of multilayer h-BN films prepared at various growth temperatures. It could be observed that the position of characteristic E_2g_ band presents a slightly shift (from 1372 to 1366 cm^−1^) with the increasing of growth temperature, indicating the increase of thickness.^24^ According to earlier reports, the full-width at half maximum (FWHM) of the Raman E_2g_ band is associated with the crystallinity of h-BN.^24–26^ The FWHM of E_2g_ phonon mode decreased with growth temperature increasing (Fig. 1c). The h-BN synthesized at 1300 °C shows the minimum FWHM value of ∼15 cm^−1^, which is much smaller than that synthesized by existing catalytic epitaxial approaches.^25^

**Fig. 2:**
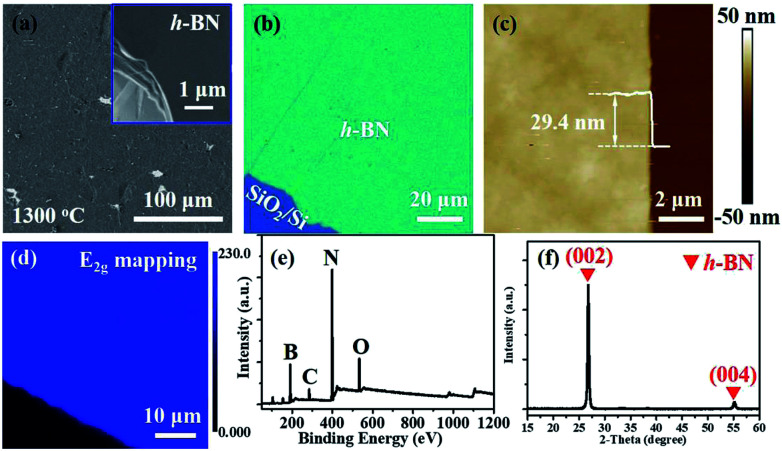
(a) SEM image of multilayer h-BN film grown on Fe–B alloy at 1300 °C. Inset shows the edge of h-BN film. (b) OM image of multilayer h-BN transferred onto SiO_2_ (300 nm)/Si substrate. (c) AFM image and corresponding height profile of h-BN film. (d) Raman mapping of E_2g_ phonon mode of h-BN film. (e and f). Survey XPS (e) and XRD (f) spectra of h-BN film.

Multilayer h-BN film obtained at 1300 °C was further characterized to analyse its morphology and crystalline quality. Continuous and uniform h-BN film grown on Fe_2_B alloy was displayed in Fig. 2a. Layered structure was verified primarily by measuring the edge of h-BN film. To further evaluate the thickness uniformity of h-BN films on Fe_2_B substrate, the as-grown multilayer h-BN was transferred on a SiO_2_ (300 nm)/Si substrate. The uniform optical contrast confirms the smooth surface and high uniformity of thickness (Fig. 2b). The thickness of the film is around 30 nm according to the height profile on the edge of h-BN (Fig. 2c). Also, Raman mapping of E_2g_ phonon mode depicts the homogeneity thickness of as-prepared h-BN multilayers (Fig. 2d). Moreover, we investigated the composition and crystal structure of h-BN film. The survey XPS spectrum of h-BN film, displayed in Fig. 2e, indicates that the stoichiometry of B and N atoms is 1 : 1.06. Further, The XRD spectrum displays sharp (0002) diffraction peak at 26.8° and (0004) diffraction peak at 55.2°, which signifies the layers of h-BN are well aligned with the *c* axis (Fig. 2f).^18^ The Inorganic Crystal Structure Database (ICSD) (h-BN, 340421) was used for phase identification. The lattice constant was calculated to be 0.33 nm, which is in good agreement with reported values.^18^

**Fig. 3:**
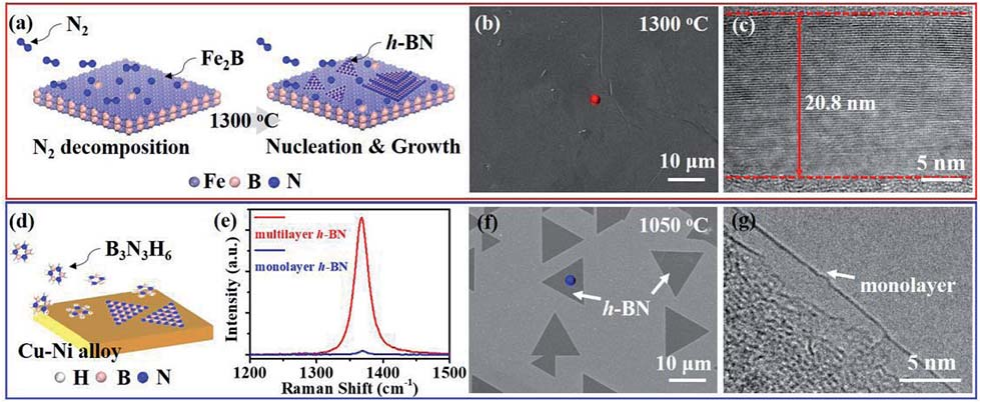
(a) Schematic of the growth process of multilayer h-BN grown on Fe_2_B alloy with N_2_ as a precursor. (b and c) Typical SEM (b) and TEM (c) images of as-grown multilayer h-BN. (d) Schematic illustration of the growth process of monolayer h-BN on Cu–Ni alloy with B_3_N_3_H_6_ as a precursor. (e) Raman spectra of multilayer (red line) and monolayer (blue line) h-BN. (f and g) Typical SEM (f) and TEM (g) images of monolayer h-BN grown on Cu–Ni alloy.

As previous reports, NH_3_·BH_3_ or B_3_N_3_H_6_ was always used to synthesize monolayer or few-layer h-BN on metal substrates.^14–17,19,21,23^ For monolayer h-BN grown on Cu–Ni alloy, B_3_N_3_H_6_ precursor undergoes decompose, polymerization and dehydrogenation on the surface of the substrate (Fig. 3d).^19^ SEM image displays the morphology of h-BN domains and TEM image signifies the monolayer nature of as-grown h-BN (Fig. 3f and g). The surfaces do not assist the synthesis of multilayer h-BN due to the self-limited growth effect when monolayer h-BN covered the Cu–Ni alloy.^19^ For multilayer h-BN grown on Fe_2_B alloy, N_2_ would firstly decompose into active nitrogen atoms with the catalysis of Fe_2_B substrate.^27^ Then, active nitrogen atoms would react with Fe_2_B to form initial B–N molecular.^18,27^ High concentration B–N molecules would segregate and induce the nucleation of h-BN isothermally. Finally, h-BN nucleus would grow and coalesce into multilayer h-BN films on the surface of Fe_2_B alloy. The controllable thickness would be realized by optimizing growth temperature or reaction time (Fig. 3a). SEM image of as-grown multilayer h-BN displays the uniform and smooth surface morphology and TEM image indicates multi-layered h-BN nanostructure (Fig. 3b and c). Further, E_2g_ phonon mode of multilayer h-BN in the Raman spectrum shows much stronger intensity than the E_2g_ band of monolayer h-BN (Fig. 3e). Comparing with the two synthesis approaches mentioned above, the success of thickness controllable growth is mainly due to the change of h-BN growth mechanism from surface-mediated growth to isothermal segregation on Fe_2_B substrate by using nitrogen as feedstock.

**Fig. 4:**
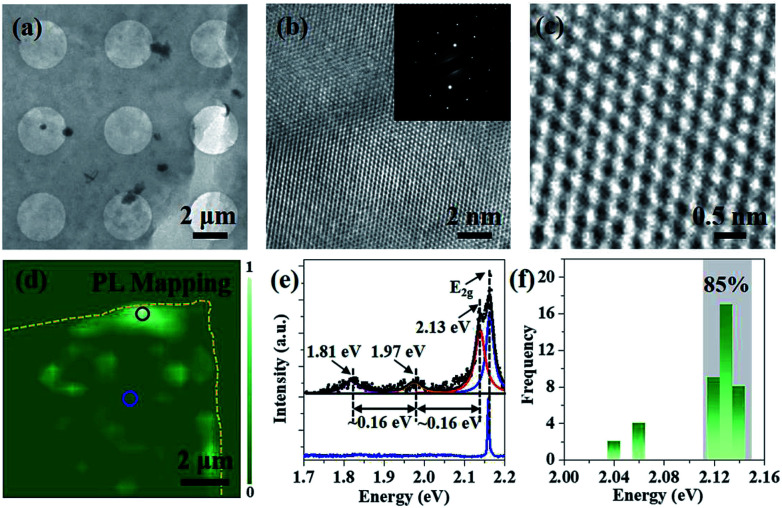
(a) TEM image of h-BN thin film suspended on Cu mesh grid. (b) High resolution TEM image of multilayer h-BN, the inset displays the SAED pattern. (c) Atomic resolution TEM image of multilayer h-BN. (d) Typical PL mapping of multilayer h-BN on Si substrate. (e) Individual PL spectra extracted from the spots selected in d. (f) Histogram of h-BN zero phonon line (ZPL) energy generated from 40 emitters from h-BN film. The shaded area highlights the range *E*_emisson_ = (2.13 ± 0.1) eV, which contains 85% of the emitters.

The crystalline quality and atomic structure of the multilayer h-BN is further characterized by TEM. The low-magnified TEM view in Fig. 4a displays continuous and uniform h-BN film suspended on Cu mesh grid. High resolution TEM image exhibits the lattice fringe of h-BN multilayers (Fig. 4b). Corresponding selected area electron diffraction (SAED) presents only one set of hexagonally arranged spots, rather than ring-shaped patterns, assuring the highly oriented stacking order.^20^ Moreover, Fig. 4c reveals a honeycomb structure with an inter-atomic distance of about 0.25 nm, in good agreement with bulk h-BN.^28^ Moreover, as earlier theoretically and experimentally analysis, point defects would induce non-resonant absorption and emission within the band gap of materials.^9,29–31^ Here, PL spectroscopy was used to investigate point defects distributions in h-BN film.^9,30,31^ PL measurement was carried out at room temperature with a 532 nm sub-bandgap excitation laser. Fig. 4d illustrates the defect distributions in h-BN flake transferred on Si substrate. Optical emissions are accumulated on the edge of as-prepared h-BN multilayers, which may be introduced by the transfer and annealing processes. Typical PL spectra were extracted and fitted by Lorentzian fitting (Fig. 4e). Three peaks centred at 2.13 eV, 1.97 eV and 1.81 eV were observed, which is consisted with zero phonon line (ZPL) and corresponding one- and two-optical-phonon sidebands (PSB), respectively.^9,30,31^ In addition, Fig. 4f displays a histogram of the emission energy values measured for 40 individual emitters. More than 85% of the emitters were measured to have a ZPL energy localized in a very narrow spectral window, (2.13 ± 0.1) eV. Such a small spectra variability of the ZPLs may be caused by strain and/or local lattice symmetry distortions.^31^ Moreover, it is observed that no optical emission was found on the large area of h-BN region, indicating the high quality of as-grown h-BN multilayers.

## Conclusion

In this work, uniform and continuous multilayer h-BN has been obtained isothermally through the reaction between Fe_2_B alloy and N_2_*via* CVD method. The thickness could be mediated by controlling diffusion rate of nitrogen in the catalyst at different growth temperatures. Raman shift at 1366 cm^−1^ and sharp (0002) diffraction peak in the XRD pattern confirm the crystallinity of h-BN. TEM measurements revealed that the h-BN has highly orientated layered lattice structure. Comparing with monolayer h-BN growth on catalytic metal surfaces, this method shows its unique mechanism for achieving multilayer h-BN, which has potential on the synthesis of other layered 2D heterostructures. Therefore, the present achievement of high-quality multilayer h-BN synthesis would give strong impact on ultrathin dielectric-, support-, or barrier-layer in particular for integrated electronics and photonics of 2D materials.

## Conflicts of interest

There are no conflicts to declare.

## Supplementary Material
